# Effects of altered gravity on growth and morphology in *Wolffia globosa* implications for bioregenerative life support systems and space-based agriculture

**DOI:** 10.1038/s41598-023-49680-3

**Published:** 2024-01-03

**Authors:** Leone Ermes Romano, Jack J. W. A. van Loon, Luigi Gennaro Izzo, Maurizio Iovane, Giovanna Aronne

**Affiliations:** 1 Department of Agricultural Sciences, University of Naples Federico II, Portici, Italy; 2 Department Oral and Maxillofacial Surgery/Pathology, Amsterdam Movement Sciences and Amsterdam Bone Center (ABC), Amsterdam University Medical Center Location VUmc and Academic Center for Dentistry Amsterdam (ACTA), Amsterdam, The Netherlands; 3 TEC-MMG-LIS Lab, European Space Agency (ESA) Technology Center (ESTEC), Noordwijk, The Netherlands

**Keywords:** Plant sciences, Light responses, Photosynthesis, Plant development

## Abstract

Understanding the response of plants to varied gravitational conditions is vital for developing effective food production in space bioregenerative life support systems. This study examines the impact of altered gravity conditions on the growth and morphological responses of *Wolffia globosa* (commonly known as “*water lentils*” or “*duckweed*”), assessing its potential as a space crop. Although an experiment testing the effect of simulated microgravity on Wolffia globosa has been previously conducted, for the first time, we investigated the effect of multiple gravity levels on the growth and morphological traits of *Wolffia globosa* plants. The plant responses to simulated microgravity, simulated partial gravity (Moon), and hypergravity environments were evaluated using random positioning machines and the large-diameter centrifuge. As hypothesized, we observed a slight reaction to different gravitational levels in the growth and morphological traits of Wolffia globosa. The relative growth rates (RGR) of plants subjected to simulated microgravity and partial gravity were reduced when compared to those in other gravity levels. The morphological analysis revealed differences in plant dimensions and frond length-to-width ratios under diverse gravity conditions. Our findings showed that Wolffia globosa is responsive to gravitational changes, with its growth and morphological adaptations being slightly influenced by varying gravitational environments. As for other crop species, growth was reduced by the microgravity conditions; however, RGR remained substantial at 0.33 a day. In conclusion, this study underscores the potential of *Wolffia globosa* as a space crop and its adaptability to diverse gravitational conditions, contributing to the development of sustainable food production and bioregenerative life support systems for future space exploration missions.

## Introduction

*Wolffia globosa*, commonly known as “*water lentils*” is a member of the *Wolffoideae* subfamily within the *Lemnaceae* family, shares similar biological traits with its *Lemnoideae* relatives: it is the smallest flowering plant and shows the fastest growth rate in the plant kingdom and lacks a pseudo root system^1,2^. Due to its rapid growth rate, high protein content, and nutritional value, it has emerged as a promising candidate for sustainable food production, particularly in agriculturally challenging regions^3–6^. This unique plant has recently garnered attention also as a potential bioregenerative life support system (BLSS) candidate^7^, offering traits that align with closed-loop, resource-efficient systems like those adopted for the ESA MELiSSA Loop^8^. Nevertheless, as for other candidate space crops^9,10^ to assess the suitability of *Wolffia’s* in space cultivation and its ability to recycle resources efficiently, further laboratory tests investigating growth, nutrition, and genetic responses under extreme conditions are fundamental^7^.

Studying the effects of various stimuli, including different gravity environments, radiations, and their interactions, on the growth and development of plants can be challenging and costly^7,11^. Space limitations within onboard test facilities, such as the International Space Station or orbiting vectors, often restrict the number of replicates, making studies under these conditions even more demanding^12–14^. However, cost-effective alternatives exist in the form of facilities that simulate microgravity and partial gravity levels by continuously altering the gravitational vector^15–17^. These facilities offer higher replicate numbers and serve as robust testbeds for experiments involving different gravity levels^18,19^.

Hypergravity, characterized by gravitational forces greater than Earth’s standard 1 g, is a significant factor in space exploration, affecting humans, and plant life^20,21^. Hypergravity is most prominent during the manuvers of take-off and landing phases of spacecraft. These brief yet intense episodes of increased gravitational force can influence various aspects of plant biology and growth^21^. Understanding how plants respond to hypergravity is essential for optimizing their cultivation in space environments and for Earth’s agriculture^22,23^. Furthermore, it provides valuable insights into the mechanisms plants employ to withstand and adapt to extreme gravitational conditions^24,25^.

Earlier studies under simulated microgravity have shown stable anatomical morphology in *Wolffia* plants^26^, possible effects of hypergravity on plant growth and reproduction have not yet been investigated. Testing plant reactions of potential space crops under varying gravity levels, including those experienced during take-off, landing, or in partial gravities, is reported as crucial^27^. Consequently, exploring the adaptability of *Wolffia* plants under different gravitational conditions significantly contributes to our understanding of their potential^7^.

Recent genome sequencing of *Wolffia australiana* has shed light on the gravity-sensing mechanisms and photomorphogenesis in *Wolffia* plants^28,29^. Due to the loss of gravity sensing genes in the sister species *Wolffia australiana*, we also hypothesised for *Wolffia globosa* a lack of the gravity sensing mechanisms, resulting in a reduced effect of the different gravity levels on the growth and morphological characteristics.

More specifically, we aim to study the effects of altered gravity conditions on the growth and morphology of *Wolffia globosa*. Leveraging the capabilities of random positioning machines and large-diameter centrifuges, we simulated microgravity, partial gravity (Moon), and hypergravity conditions to comprehensively explore the impact of varying gravitational environments on this plant species. These machines allow us to generate and study a range of gravity conditions, thereby elucidating *Wolffia globosa*'s responses to these diverse gravity environments and assessing its potential utilisation in space-based food production systems.

## Materials and methods

### Plant material and cultivation

Plants of *Wolffia globosa* (9910) have been provided by Prof. Klaus Apperoth from the Department of Plant Physiology of the University of Jena, Germany. Upon receive, plant material was surface sterilised with 0.3% bleach/water solution for 5 min^30^. After 14 days from disinfection, plants were subcultured for 30 days in N-medium^30^ under axenic conditions. Therefore, plant material has been transferred under a laminar flow hood in sets of 6-well plates, each filled with 5 ml of N-medium solution and sealed with micropore® tape. Before the experimental run, plants were acclimatised for 24 h at 30 °C. After acclimatisation, an average of 20 ± 4 fronds were transferred in each well of a 6-multiwell plate previously filled with 5 ml of N-medium and 0.8% of Agar to achieve a semi-solid substrate. The experiment was conducted at 30 ± 0.5 °C average temperature with a photoperiod of 16/8 h light/dark and a total Photon Flux Density (PFD) of 72.37 ± 10.30 μmol m^−2^ s^−1^ for 168 h^2^. Carbon dioxide concentration has been monitored throughout the experiment, resulting in an average concentration of 303 ± 34 ppm.

### Hardware description and dimensions

**Figure 1:**
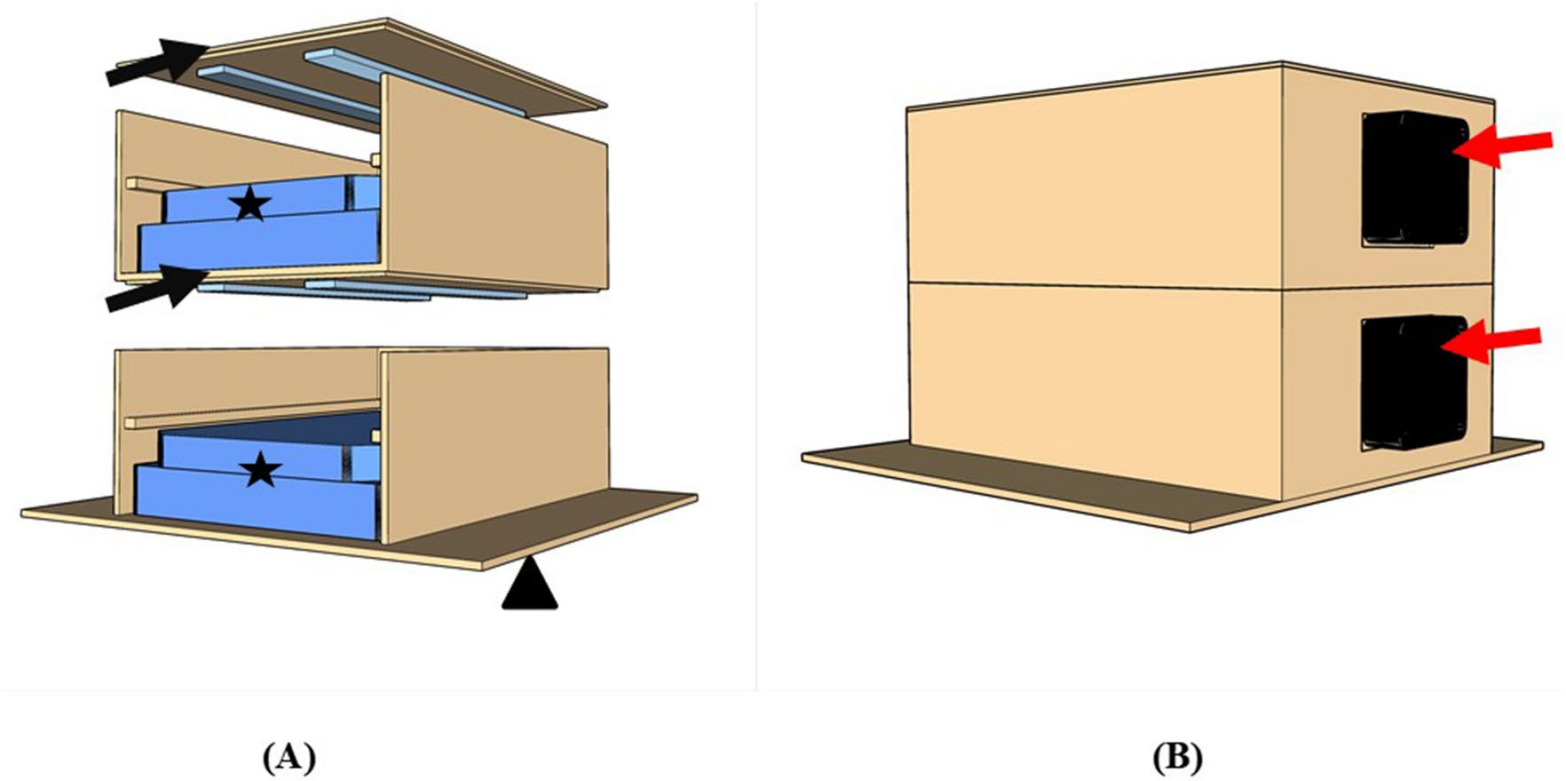
Experimental hardware: (**A**) In the Front view, the black arrows show the LED light strips, the star shapes marks the biological containers (multiwell plates), and the triangular shape marks the base plate of the experimental hardware. (**B**) Assembled hardware from a back view, the red arrows points at the 30 mm fan re-circulating air within the experimental hardware.

Experimental hardware has been developed to hold two multiwell plates in a setup that minimises the gravity gradient between the upper and lower multiwell plates. Furthermore, the experimental hardware had to be designed to fit in most test facilities, setting the lower constraints to an overall dimension of 15*15*15 cm of the smallest Random Positioning Machine (RPM) available.. Considering these constraints, we designed and developed the experimental hardware using the free available software SketchUp® (Fig. 1). Due to the constraints described before, multiwell plates had to be stacked vertically. This setup minimises the distance of the two centres of mass of both multiwells so that the difference in acceleration can be neglected. The experimental hardware has been equipped with LED white light (Goming LTD) to ensure proper plant development.

Furthermore, 3 cm 5 V fans have been added to the experimental hardware to increase uniformity between the temperature inside and outside the experimental hardware (Fig. 1). The prototype of the experimental hardware has been built in plywood. Plywood has been laser cut, and parts have been glued in place with vinyl glue. The template for laser cutting can be found in supplementary material Appendix A. Nevertheless, the experimental hardware can be 3D printed in any plastic material, and information for the 3D model can be found on the directory: GitHub.

**Figure 2:**
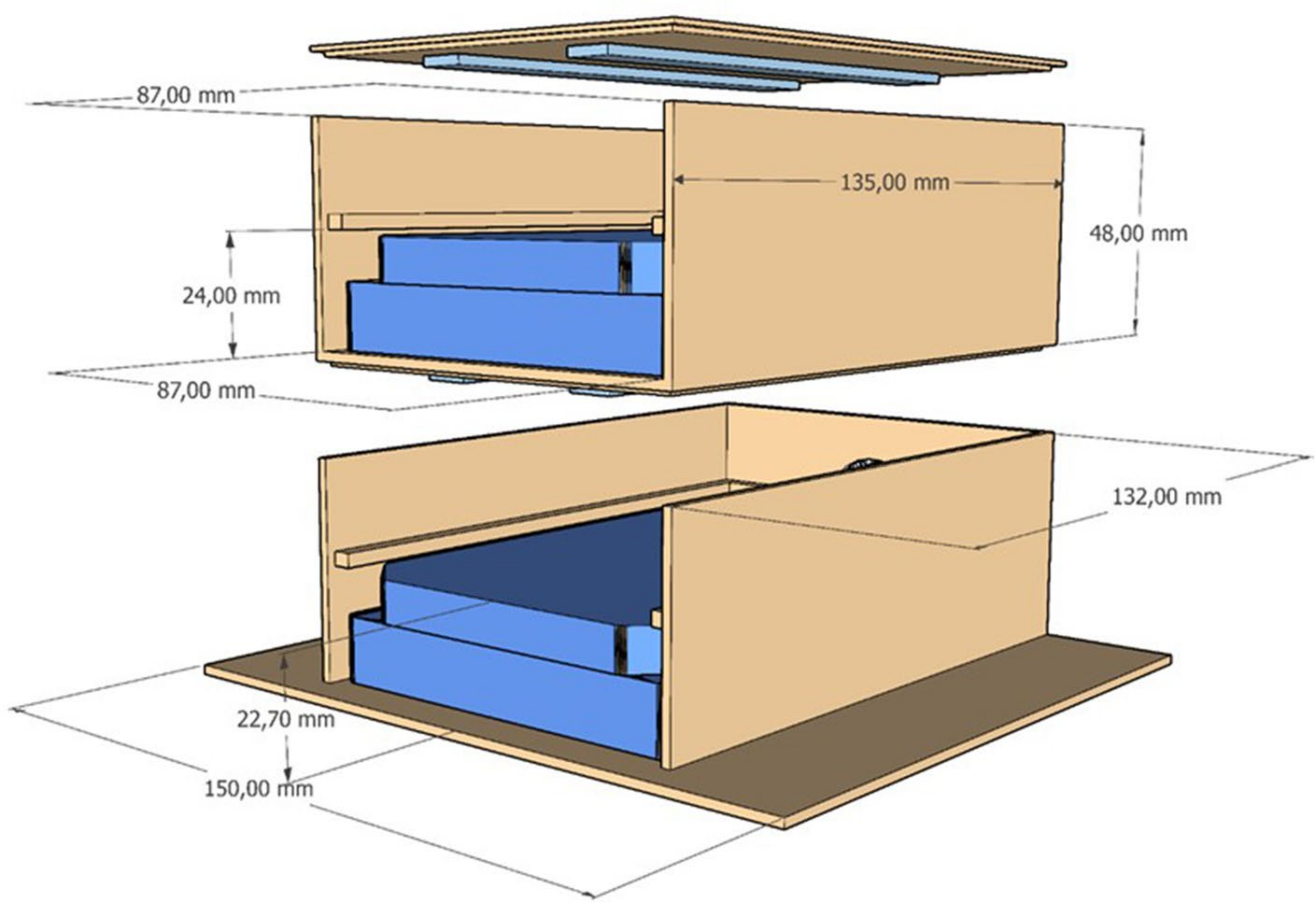
Experimental hardware, with the exact dimensions of every component.

The inner dimension of each experimental hardware is designed to fit a multiwell plate of standard dimensions (12.7 × 8.5 × 2.2 cm). From the multiwell lid to the lighting system, a fixed distance of 2.0 cm has been set up to guarantee optimal light intensity for plant growth. The experimental hardware's design and dimensions are reported below (Fig. 2).

### Lighting system

**Figure 3:**
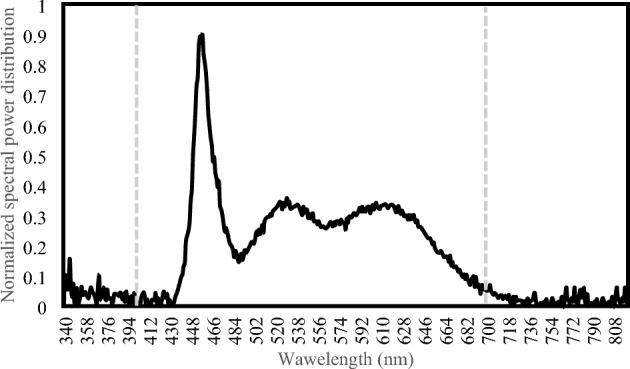
Emission spectra of the LED stripe used in the experiment. The graph shows emission spectra regarding light quality and quantity of the light source. Dotted lines limit the photosynthetically active radiation (PAR).

Light treatment has been achieved utilising two 10 cm 5 V LED stripes illuminating each multiwell plate (Fig. 1). Via USB connector, the light was connected to a Wi-Fi USB switch that controlled the lighting system and photoperiod to a 16/8 light/dark cycle. Light emission spectrum has been determined with a spectroradiometer (SS-110, Apogee Instruments Inc.) measuring in six different positions within the experimental hardware (N = 6) in a wavelength range of 340–820 nm. A more detailed composition of the light spectrum is described in Table 1 and Fig. 3.

**Table 1:** Light measurements.

	Total PFD	PPF	YPF	PPE	R:FR
Average	72.37	70.23	59.50	0.84	7.54
Stdev.	10.30	9.99	8.47	0.00	0.01

The following parameters are expressed in total photon flux density (PFD) (μmol m^−2^ s^−1^), photosynthetic photon flux (PPF) (μmol s^−1^), yield photon flux (YPF) (μmol s^−1^), and photosynthetic photon efficacy (PPE); red-to-far red ratio (R/FR).

### Gravity treatments

**Figure 4:**
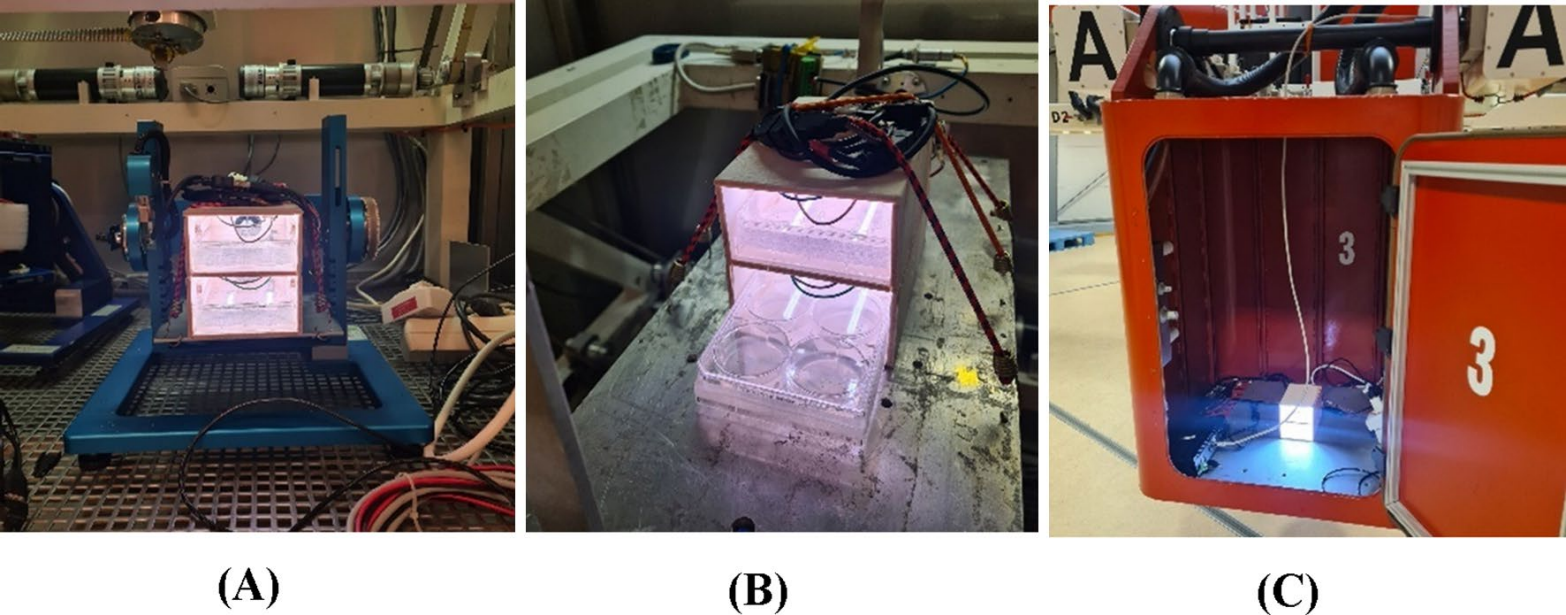
Experimental hardware implementation. The different images show the implementation of the experimental hardware in the different gravity treatments. (**A**) RPM simulated partial gravity (Moon) (**B**) Simulated microgravity (sim-µg) (**c**) LDC gondola implemented with the experimental hardware within the LDC.

The study encompassed four different gravity treatments: simulated microgravity, simulated partial gravity (Moon), and two levels of hypergravity (2*g* ad 4*g*). In addition, a control group under Earth gravity conditions was included for comparison (Fig. 4).

Simulated microgravity (sim-µg) was achieved using an RPM (Random Positioning Machine, original Dutch Space Leiden, NL; currently Yuri Space, Meckenbeuren, DE) that allowed the plants to be positioned within 10 cm from the centre of rotation. The RPM was set to a maximum random speed of 60 °/s, minimising residual acceleration^31^. RPM was also set to random interval and random direction.

Simulated Partial gravity (Moon) (sim-Moon) was simulated using a similar RPM, which was running a different program which does not provide a complete random rotation during the experiment but generates vector orientations and values that are biased such that the averaged resulted gravity vector simulates the Moon. This proprietary software generated motion can enabled the examination of plant responses to partial gravity ranging from nearly zero to 0.9 g levels^27^.

Hypergravity conditions were obtained using the large-diameter centrifuge (LDC)^32^. Two hypergravity levels were tested: 2*g* and 4*g*, corresponding to twice and four times the gravitational force on Earth, respectively. These conditions allowed investigation of how *Wolffia globosa* responded to increased gravitational forces.

A control group was included, where plants were grown under normal gravity conditions (1*g*) in a static gondola. This control group served as a baseline for comparing the growth and morphology of *Wolffia globosa* under different gravity treatments.

The experimental setup and the number of replicates for each treatment are summarised in Table 2:

**Table 2:** Gravity treatments and replicates.

Treatment	Label	Facility	Replicates	Note
Control (1*g*)	1 g	Static Gondola	12	Plants grown under normal gravity conditions
Simulated Microgravity	sim-µg	RPM	12	Plants exposed to simulated microgravity
Partial Gravity (Moon)	sim-Moon g	RPM	12	Plants exposed to simulated partial Moon gravity
Hypergravity 2*g*	2 g	LDC	12	Plants exposed to a gravity level of 2*g*
Hypergravity 4*g*	4 g	LDC	12	Plants exposed to a gravity level of 4*g*

### Data collection and analysis

We collected data by imaging the multiwell plates at two-time points: the beginning of the experiment (t0) and after 168 h (t168).

Two imaging setups were used:

Camera Setup: a Sony Alpha II camera with an 18–70 mm objective was fixed to a camera stand. It captured top-down images of the multiwell plate placed on a lighting table with a white semi-transparent Polyvinyl chloride panel illuminated by an LED light panel, ensuring uniform lighting conditions. The images thaken with this setup were used to evaluate growth. Morespecificaly, the relative growth rate (RGR) was calculated by comparing the area occupied by the plants in pixels at the start of the experiment (t0) and after 168 h (t168). Doubling time (DT) was determined based on RGR.

Stereo Microscope Setup: detailed morphological analysis was performed using a Leica® MZ8 Stereo zoom Microscope. Images representing replicates for each treatment were captured. Images acquired with the steromicroscope were used to evaluate the morphological traits of *Wolffia globosa* were compared among gravity treatments. Morphological traits, such as the dimensions of the long and short axes of the mother fronds, were measured using Fiji® software. Ratios between the long and short axes of the mother fronds were also calculated to assess frond roundness.

**Figure 5:**
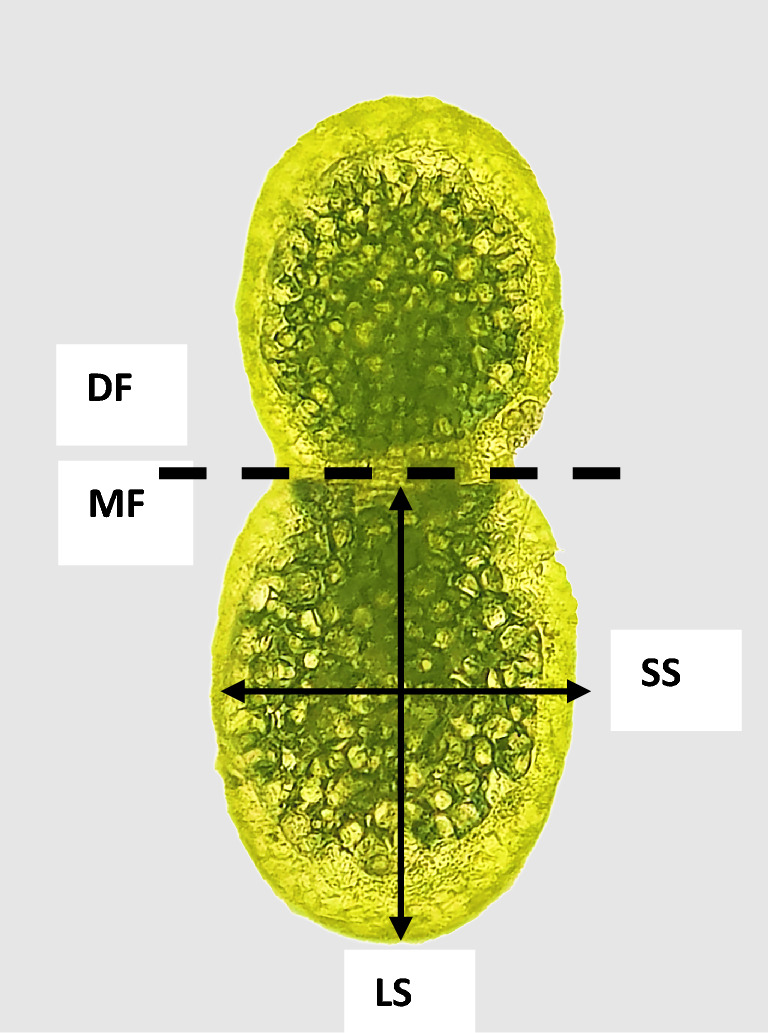
*Wolffia globosa* plant. The dotted line divides the daughter frond (DF) from the mother frond (MF). The (LS) arrow shows the long side, and the (SS) arrow shows the short side.

Image analysis has been performed on the images gathered during the experimental run. Two types of analysis have been conducted: (1) Analysis of the growth parameters (RGR and DT) (2) Analysis of the morphological differences. RGR has been calculated by comparing the area expressed in pixels occupied by the plant at two-time intervals (N = 12). Images were analysed using the ilastik® software described by Romano et al.^33^. We calculated DT based on RGR as described by Naumann^34^. To perform morphological comparison among treatments, one hundred mother fronds have been randomly selected throughout the different wells of the same multiwell plate within the same treatment. For coherent results, a phenological standard was determined. We have measured only mother fronds that had a well-formed daughter frond still attached. Morphological traits have been evaluated by measuring the long and short side of the mother frond. Long and short side have been always measured as shown in Fig. 5. Furthermore, we have evaluated the existing ratios with respect to the long side between these two measurements (N = 100)^26^ (Fig. 5). Measurements have been taken utilising Fiji® software.

Image analysis data were processed and analysed using SPSS Statistics ver. 21 (IBM Corp.). Differences in growth and morphological traits of *Wolffia globosa* among gravity treatments was assessed by one-way ANOVA (*p* < 0.05). Pairwise comparisons were performed using Tukey's post-hoc test (*P* < 0.05) to identify differences among individual treatments.

### IUCN policy statement

Experimental studies and field research on plants (cultivated or wild), including the collection of plant material, must comply with relevant institutional, national and international guidelines and legislation. We will strictly adhere to the IUCN Policy Statement on Research on Endangered Species and the Convention on Trade in Endangered Species of Wild Fauna and Flora. Prof. Klaus Apperoth provided specimens in this utilised in this study from the Department of Plant Physiology of the University of Jena, Germany and are available in the collection of the up mentioned university. We confirm compliance with the IUCN policy for plant.

## Results

### Relative growth rate (RGR)

**Figure 6:**
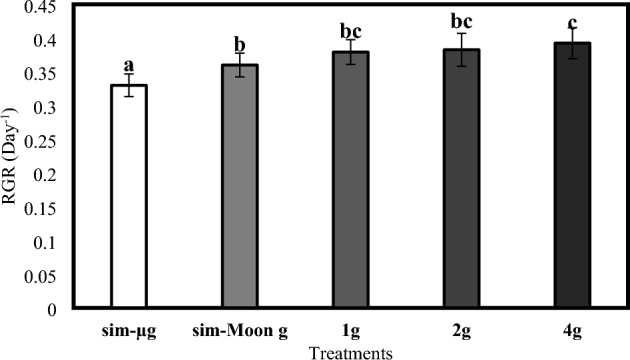
Observed RGR across the different treatments. Graph shows the relative growth rate (RGR) resulting from the different gravities levels. Data refer to means ± SD of the means (N = 12). The letters on top of the bars describes the homogenous subsets calculated with the Tukey's post-hoc analysis.

The analysis of variance (ANOVA) revealed a significant difference (F = 16.47, *p* < 0.001) for the RGR among the different gravity treatments. The Eta-squared and Epsilon-squared effect sizes indicated a large effect of the treatments on the RGR (Eta-squared = 0.54, Epsilon-squared = 0.51). The post hoc 'Tukey's test, conducted to determine the specific differences between the treatments, showed that the simulated microgravity treatment (sim-µg) (mean difference = 0.33, *p* < 0.001) had a significantly lower RGR (Fig. 6) compared to the control treatment (1 g) (mean difference = 0.38 ± 0.02). The simulated Moon partial gravity treatment (sim-Moon g) also exhibited a lower RGR than the control treatment, but the difference was not statistically significant (mean difference = 0.36, *p* = 0.25).

**Figure 7:**
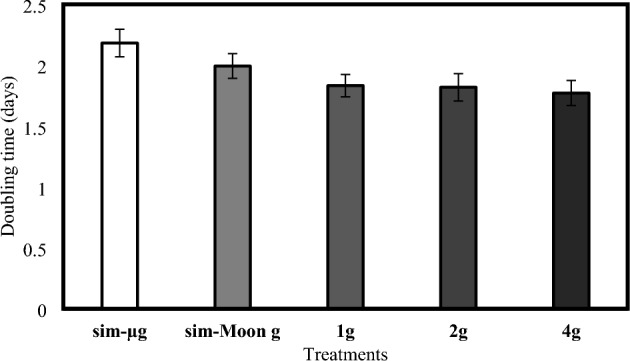
Observed DT across the different treatments. The graph shows the calculated doubling time-based on the RGR expressed in the different gravity levels. Data refer to means ± SD of the means N = 12.

In contrast, hypergravity treatments (2 g and 4 g) showed higher RGR compared to the control treatment. However, there was no significant difference between the control and 2 g hypergravity treatments (mean difference = 0. 38, *p* = 0.99). The 4 g hypergravity treatment (4 g) showed the highest RGR among all treatments, however the difference was not statistically significant compared to the control treatment (mean difference = 0.39, *p* < 0.50). Furthermore, the doubling time is reported. The sim-µg treatment exhibited the longest doubling time of 2.18 days, followed by the sim-Moon g, 1 g, 2 g, and 4 g treatments with doubling times of 1.99, 1.83, 1.82, and 1.82 days, respectively (Fig. 7).

### Morphological difference

**Figure 8:**
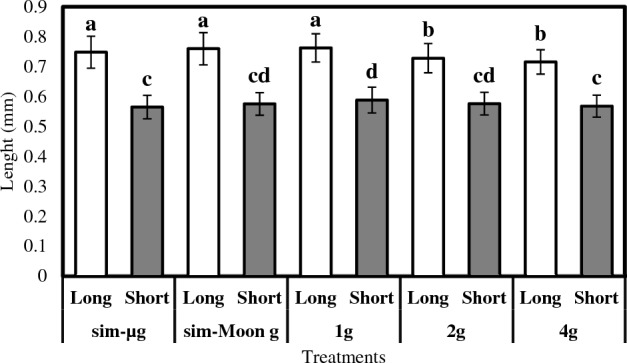
Comparison between average long and short sides across different treatments. Graphs show the average length in mm of the long and short sides of different mother fronds across different gravity treatments. Data refer to means ± SD of the means, N = 100. The letters on top of the bars describe the homogenous subsets calculated with Tukey's post-hoc analysis.

Analysis of the morphological characteristics of *Wolffia globosa* fronds revealed differences in frond length among the gravity treatments. Resulting in a variation in the length of both short and long axes. Results of the ANOVA for the long side length showed a significant difference among gravity treatments (F = 17.34, *p* < 0.001). Results revealed that the fronds in the 1 g treatment exhibited the longest length (mean = 0.76 mm), followed by the sim-Moon g treatment (mean = 0.76 mm), the sim-µg treatment (mean = 0.75 mm), the 2 g treatment (mean = 0.73 mm), and the 4 g treatment (mean = 0.71 mm) (Fig. 8). Similarly, for the short side length, a significant main effect of gravity treatment was found (F = 5.349, *p* < 0.001). Post hoc tests showed that the fronds in the sim-µg treatment displayed the shortest length (mean = 0.56 mm), followed by the 4 g treatment (mean = 0.57 mm), the sim-Moon g treatment (mean = 0.57 mm), the 2 g treatment (mean = 0.58 mm), and the 1 g treatment (mean = 0.59 mm). These results suggest that the gravity environment significantly affects the morphological characteristics of Wolffia globosa fronds, resulting in shorter (long axis) plants for those that exhibited the highest growth and vice versa.

**Figure 9:**
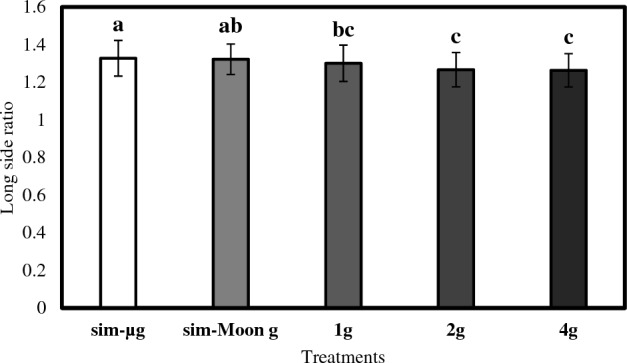
Long side ratio of the measured mother fronds across different treatments. Data refer to means ± SD of the means, N = 50. The letters on top of the bars describes the homogenous subsets calculated with the Tukey’s post-hoc analysis.

Analysis of the ratio in relation to the long side of *Wolffia globosa* fronds revealed significant differences among the different gravity treatments (F = 11.162, *p* < 0.001). Post hoc tests reported that the sim-µg treatment exhibited the highest ratio value (mean = 1.33), followed by the sim-Moon g treatment (mean = 1.32), the 1 g treatment (mean = 1.30), the 2 g treatment (mean = 1.27), and the 4 g treatment (mean = 1.26). These results suggest that the low gravity environment (sim-µg and sim-Moong) significantly impacts the ratio in relation to the long side of *Wolffia globosa* fronds (Fig. 9).

## Discussion

The findings of this study shed light on the response of *Wolffia globosa* to altered gravitational conditions. While the results revealed some variations in growth and morphological traits among the different gravity treatments, these observations present exciting opportunities for further exploration and potential applications.

In the realm of plant cultivation for space exploration (on orbital platforms, the Moon, and Mars), a more profound comprehension of plant reactions to altered gravitational conditions is essential^16^. Previous studies have shown various effects of microgravity on the growth of several plant species, but research on the effects of partial gravity and hypergravity on plants has been limited^15,18^. The aid of facilities such as RPMs and LDC can be the perfect testing ground before experimenting with true microgravity^18,19^ and testing the effect of different gravity levels on the performances of novel space crop species^7^.

Plants grown under microgravity conditions manifest a slower growth rate compared to those grown under Earth’s gravity^35^. Results from our investigation show that simulated microgravity and simulated Moon gravity treatments resulted in slightly lower growth rates than the control treatment (Fig. 5). Although these differences, it is important to note that the relative growth rates observed in these treatments were still substantial, with the simulated microgravity treatment exhibiting a relative growth rate of 0.33 per day and the simulated Moon partial gravity treatment exhibiting a relative growth rate of 0.36 per day while it was 0.38 per day in the 1 g control plants. In addition, although reduced by the altered gravity conditions, growth rates of the *Wolffia* plants remain higher than those typically observed in other higher plant species^36^. Results from past experiments show the relative growth rate over a span of 40 days of lettuce grown under simulated microgravity, yielding a rate of 0.08/day^37^.

The effects of altered gravity, especially microgravity (whether real or simulated), on higher plant species have been the subject of numerous scientific studies^38^. *Wolffia globosa* is the smallest higher plant, and its morphology is entirely distinct from other higher plants^39^. It doesn’t exhibit the typical morphology of roots, stems, and leaves but only has a frond that cannot be associated with any of the three main organs^39^. The results of previous studies on the effects of altered gravity on plant growth are not directly comparable to this species. Most of the research conducted focuses on the interactions between microgravity and roots^10–12,14,21^. *Wolffia globosa* is famously known as a rootless plant^39^. To further complicate matters, their rootless nature makes comparison with what has been studied so far challenging. Additionally, while microgravity has often been reported as a limiting growth factor for plants^40–42^, results from tests performed under hypergravity are disagreeing. Studies on the nucleolar activity or on the elongation of hypocotyls in both *Arabidopsis thaliana* and *Cucumis sativus*, showed a decrease in growth rates^43,44^. At the same time, other results suggest that hypergravity might not affect growth^45^. In our experiment, the 2*g* and 4*g* hypergravity treatments manifest an increasing (although not statistically significant) trend in growth rates compared to the control treatment (Fig. 6). This possible positive response to hypergravity treatments opens up interesting possibilities for future studies and potential applications in space agriculture but requires new ad hoc tests, eventually applying higher levels of hypergravity to amplify the phenomenon. In such a scenario, hypergravity conditions could be leveraged to enhance plant productivity and biomass accumulation in controlled environments, such as space stations.

Considering that morphology plays a crucial role in plant growth, survival, and productivity^46^, we investigated the effect of different gravitational treatments on the shape of *Wolffia globosa* frond. Moreover, results revealed significant differences among the treatments for both the long and short sides. Worth mentioning that such an effect occurred on plants that can be considered genetically identical.

The study findings revealed that the fronds of *Wolffia globosa* were most extended in the 1 g treatment, both on the front and back sides. Subsequently, the sim-Moon g, sim-µg, 2 g, and 4 g treatments followed in terms of frond length. These results suggest that both simulated microgravity and simulated Moon’s partial gravity have don’t have an impact on frond elongation. Furthermore, when analysing the ratio in relation to the longer side, significant differences were observed among the gravity treatments, indicating distinct frond shapes. The sim-µg treatment resulted in the most elongated frond shape, followed by the sim-Moon g, 1 g, 2 g, and 4 g treatments. These findings provide compelling evidence supporting the significant influence of gravity on the morphological characteristics of Wolffia globosa fronds. The observed variations in frond length and shape among the treatments underscore the role of gravity in shaping plant growth and development. Specifically, the sim-µg treatment consistently induced the most pronounced changes, suggesting its potential as a key factor in determining frond morphology. These results contribute to our understanding of how different gravity environments impact the growth and shape of *Wolffia globosa*, highlighting the importance of considering gravity conditions in cultivating and manipulating these plants for various space applications. Understanding the underlying mechanisms driving these morphological changes could provide valuable insights into the plant's ability to perceive and respond to gravitational cues.

The adaptability of *Wolffia globosa* to altered gravity conditions suggested by our data hints at the potential use of plants from the genus *Wolffia* in space agriculture. These plants, characterised by rapid growth and high nutrient content, are strong candidates for food production in space, where resource constraints are critical^7,13^. Our findings further appraise the possible use of *Wolffia globosa* for space exploration and the establishment of sustainable agriculture in extraterrestrial environments. The robust growth and adaptability to altered gravity conditions can be added to the small size, rapid growth rate, and high nutrient content, confirming that this genus deserves consideration for cultivation in limited space and resource-constrained environments^7^.

Although *Wolffia globosa* exhibited a reduced growth rate in our experiment under simulated microgravity, previous research has seen growth enactment of *Wolffia globosa* under simulated microgravity^26^. As already mentioned by the authors of the up-mentioned work, the enhanced growth might be linked to better utilisation of dissolved oxygen and organic substances in the growth medium due to the type of cultivation system they have employed^26^. The varying responses observed among different gravity treatments in our experiment could be attributed to the interplay between genetic variability within the Lemnaceae family and the specific environmental conditions experienced by each individual plant. Notably, the genus *Wolffia* comprises numerous species with significant genetic diversity. Testing the effects of altered gravity on only one clone of *Wolffia globosa*, as done in our experiment, provides a valuable starting point but may not fully capture the entire range of responses within the genus *Wolffia*. Further investigations involving multiple *Wolffia* species and clones are warranted to explore the full extent of the species' reaction to altered gravity and better understand the role of genetic variability in shaping their growth patterns.

The recent sequencing of the genome of *Wolffia australiana* has provided valuable insights into the genetic characteristics of *Wolffia* species^47^. One intriguing finding is the absence of gravity-sensing genes commonly observed in higher plants. More specifically, new findings have shed light on the missing LAZY proteins in *Wolffia australiana*^28^. This genetic difference suggests that *Wolffia* species have evolved alternative mechanisms to perceive and respond to gravity cues, which may explain the distinct growth patterns exhibited under altered gravitational conditions. Furthermore, to some degree, the enhanced growth rate seen under the hypergravity treatment could be attributed to gravity resistance genes such as PTH2^48^. Our results partially confirmed the expected limited reaction to gravity also of *Wolffia globosa*. The adaptability of *Wolffia globosa* to different gravitational conditions supported the hypothesis that the loss of gravity-sensing genes reported in the sister species *Wolffia australiana* might also occur in *Wolffia globosa*. The slight variations in growth rates and morphological traits observed among the treatments open up interesting avenues for future research and potential applications in space agriculture.

In addition to its implications for space agriculture, this research also contributes to our understanding of fundamental plant biology. The findings of our experiment might encourage further investigations to clarify the evolutionary pathways that shaped the peculiar adaptation to gravity associated to a return to an ancestral aquatic environment of *Wolffia* genus.

### Supplementary Information

Supplementary Information 1.

Supplementary Information 2.

Supplementary Information 3.

Supplementary Information 4.

Supplementary Information 5.

Supplementary Information 6.

Supplementary Information 7.

Supplementary Information 8.

## Data Availability

The raw data supporting the conclusions of this article will be made available by the authors, without undue reservation.
